# Specific Interaction of DARPin with HIV-1 CA_NTD_ Disturbs the Distribution of Gag, RNA Packaging, and Tetraspanin Remodelling in the Membrane

**DOI:** 10.3390/v14040824

**Published:** 2022-04-15

**Authors:** Sutpirat Moonmuang, Rawiwan Maniratanachote, Paninee Chetprayoon, Kanokporn Sornsuwan, Weeraya Thongkum, Koollawat Chupradit, Chatchai Tayapiwatana

**Affiliations:** 1 Center of Biomolecular Therapy and Diagnostic, Faculty of Associated Medical Sciences, Chiang Mai University, Chiang Mai 50200, Thailand; sutpirat_m@cmu.ac.th (S.M.); kanokporn_sornsuwan@cmu.ac.th (K.S.); weeraya.t@cmu.ac.th (W.T.); koollawat.chu@mahidol.ac.th (K.C.); 2 Department of Medical Technology, Division of Clinical Immunology, Faculty of Associated Medical Sciences, Chiang Mai University, Chiang Mai 50200, Thailand; 3 Toxicology and Bio Evaluation Service Center (TBES), National Science and Technology Development Agency (NSTDA), Pathum Thani 12120, Thailand; rawiwan.man@nstda.or.th (R.M.); paninee.che@nstda.or.th (P.C.); 4 Center of Innovative Immunodiagnostic Development, Faculty of Associated Medical Sciences, Chiang Mai University, Chiang Mai 50200, Thailand; 5 Siriraj Center for Regenerative Medicine, Faculty of Medicine Siriraj Hospital, Mahidol University, Bangkok 10700, Thailand

**Keywords:** HIV-1, Gag polyprotein, virus assembly inhibitor, ankyrin, tetraspanin

## Abstract

A designed repeat scaffold protein (Ank^GAG^1D4) recognizing the human immunodeficiency virus-1 (HIV-1) capsid (CA) was formerly established with antiviral assembly. Here, we investigated the molecular mechanism of Ank^GAG^1D4 function during the late stages of the HIV-1 replication cycle. By applying stimulated emission-depletion (STED) microscopy, Gag polymerisation was interrupted at the plasma membrane. Disturbance of Gag polymerisation triggered Gag accumulation inside producer cells and trapping of the CD81 tetraspanin on the plasma membrane. Moreover, reverse transcriptase-quantitative polymerase chain reaction (RT-qPCR) experiments were performed to validate the packaging efficiency of RNAs. Our results advocated that Ank^GAG^1D4 interfered with the Gag precursor protein from selecting HIV-1 and cellular RNAs for encapsidation into viral particles. These findings convey additional information on the antiviral activity of Ank^GAG^1D4 at late stages of the HIV-1 life cycle, which is potential for an alternative anti-HIV molecule.

## 1. Introduction

The human immunodeficiency virus-1 (HIV-1) group-specific antigen (Gag) polyprotein is the precursor of capsid (CA) protein (the target of Ank^GAG^1D4) and is encoded by genomic RNA (gRNA; also known as full-length (FL) RNA). Gag plays an important role in viral assembly and RNA recruitment for HIV-1. Gag contains four major domains: matrix (MA), CA, nucleocapsid (NC), and p6, in addition to two spacer peptides, SP1 and SP2. Gag is myristoylated at the N-terminus of MA and contains a highly basic region (HBR) involved in targeting Gag to phosphatidylinositol 4,5-bisphosphate, PI (4,5) P2 and anchoring it to the inner leaflet of the host cell plasma membrane (PM), where viral assembly takes place [1,2].

The Gag precursor promotes HIV-1 FL RNA dimerisation in the cytoplasm and specifically targets the dimeric FL RNA to virus-assembly sites at the PM [3,4]. The virus-assembly site is composed of thousands of Gag polyprotein molecules, hundreds of Gag–polymerase (Pol) precursor proteins, 8–10 envelope (Env) protein trimers, and dimeric FL RNA [5,6]. Subsequently, the Gag polyprotein recruits the cellular endosomal sorting complexes required for transport (ESCRT) machinery for budding and membrane scission for viral particle egress [7].

After synthesis, the primary FL HIV-1 transcript mediates several key roles in viral replication. Not only being a precursor of spliced mRNA synthesis, it also acts as a template for viral protein production and as a genome incorporated into viral progeny [8]. The Gag precursor protein must select FL HIV-1 RNA from numerous cellular and viral spliced RNAs, including multiply spliced (MS) and singly spliced viral mRNAs (with env mRNA being the major determinant) [9]. However, spliced viral RNAs can be selected due to the presence of an internal loop and lower part of stem-loop 1 (SL1): in that context, the Gag precursor protein recognises FL HIV-1 RNA with higher affinity than spliced RNAs [10]. In addition to viral RNAs, retroviruses package significant amounts of cellular RNAs randomly package significant amounts of cellular RNAs including Pol-III RNA species such as 7SL and U6 RNAs [11,12]. Indeed, cellular 7SL RNA, a component of signal-recognition particles (SRPs) involved in protein translocation across the endoplasmic reticulum [11,12] and U6 spliceosomal RNA [11] are both enriched in HIV-1 particles.

During HIV-1 assembly and release, cellular tetraspanins are recruited by Gag to egress sites [13]. Tetraspanins belong to a large family of membrane glycoproteins characterised by four transmembrane proteins that are widely expressed in human cells. They play many essential roles in cellular and infectious processes [14,15]. Tetraspanins can form dynamic networks of interacting proteins at the PM by interacting with each another and with other transmembrane proteins, and such networks are referred to as tetraspanin-enriched microdomains (TEMs) [16,17]. Data from several studies showed that tetraspanins (mostly CD9, CD63, CD82, and CD81) can interact with HIV-1 Gag and Env at the PM [13,18,19], even in endosomal HIV-1-containing compartments or multivesicular body (MVB)/late endosomes in macrophages [20,21]. In addition, after Gag accumulation at the budding site, CD81 and CD9 expression on the PM decreased [14] and were associated with released virions [18,22].

Regarding the therapeutic arsenal against HIV infection, peptide and protein candidates for HIV-1 therapy have been developed and HIV-1 replication can be successfully blocked by targeting Gag proteins, as reviewed previously [23]. However, naturally occurring Gag polymorphisms have been reported that can severely compromise the susceptibility of HIV-1 to the inhibitors. The CA protein was shown to contain the most conserved region in the Gag polyprotein [24]. Inhibitors targeting Gag have been improved over the years by identifying several new CA inhibitors. Small molecules or peptide inhibitors were designed to target many sites of CA, for instance, (i) small molecules targeting the N-terminal domain of HIV-1 CA (CA_NTD_) such as CAP-1 [25], benzodiazepines [26], PF74 [27], and pyrrolopyrazolones [28]; (ii) small molecules and peptides targeting the C-terminal domain of HIV-1 CA (CA_CTD_) such as CAI peptide [29], CAC-1 peptide [30], glycodeoxycholate [31], and ebselen [32]; and (iii) small molecules targeting CA-SP1, which is a less-conserved region in HIV-1 [33], such as bevirimat [34]. A maturation inhibitor was tested in a phase-II clinical trial, although testing was terminated because the inhibitor caused SP1 polymorphisms [35]. Viral variants resistant to the pyridone-based compound PF-46396, belonging to a second class of maturation inhibitors, were also reported [36]. Nevertheless, none of these inhibitors were reported to have a collateral effect on membrane remodelling.

Interestingly, a designed ankyrin repeat protein (DARPin), known as Ank^GAG^1D4, was selected from phage-displayed ankyrin libraries based on its binding to HIV-1 CA_NTD_ [37], which is the most conserved region in HIV-1 CA [24]. The distinctive property of Ank^GAG^1D4 is the ability to inhibit HIV-1 replication [37,38,39]. Viral production and loads decreased significantly in HIV-1-infected SupT1 cells stably expressing Ank^GAG^1D4 or in culture supernatants, respectively [38]. Ank^GAG^1D4 also inhibited HIV-1 replication in primary CD4+ cells [37]. Recently, molecular dynamics (MD) simulations and nanoparticle tracking analysis (NTA) elucidate the interaction between Ank^GAG^1D4 and HIV-1 CA [40]. Despite the in silico study supporting anti-HIV-1 activity of Ank^GAG^1D4, the molecular mechanisms whereby Ank^GAG^1D4 inhibits HIV-1 replication still remain undeciphered. In this study, we investigated the impact of Ank^GAG^1D4 on the late stages of viral replication: Gag polymerisation, RNA packaging, and CD81-based tetraspanin membrane remodelling. Our data indicated that Ank^GAG^1D4 disturbed Gag distribution in PM, resulting in reduced HIV production.

## 2. Materials and Methods

### 2.1. Cell Culture and Plasmids

HEK293T cells and HeLa cells were maintained in Dulbecco’s modified Eagle’s medium (DMEM) supplemented with 2 mM of L-glutamine (Gibco, Waltham, MA, USA), 100 U/mL of penicillin (Gibco, Waltham, MA, USA), 100 mg/mL of streptomycin (Gibco, Waltham, MA, USA), and heat-inactivated fetal calf serum (10% *v*/*v*). cells were cultured in humidified 5% CO_2_ atmosphere incubator at 37 °C.

The pCEP4 vector (Invitrogen, Waltham, MA, USA) for constitutively expressing N-terminus myristoylated (Myr (+)) ankyrin, including Myr (+) Ank^GAG^1D4 and non-binder Myr (+) Ank^A3^2D3, was constructed and described previously [37]. Each ankyrin was fused with a green fluorescent protein (GFP) allowing the observation under fluorescent microscopy and analysis by flow cytometry.

A lentiviral vector CGW, a third-generation lentiviral vector, used as the backbone vector to transfer the genes for molecular scaffolds Myr (+) Ank^GAG^1D4 or Myr (+) Ank^A3^2D3 into target cells, was constructed and described previously [39]. Tagging of enhanced-green fluorescent protein (EGFP) on C-terminus of ankyrin supported the detection of ankyrin expression inside the cells. 

The pFC14K HaloTag^®^ CMV Flexi vector (Promega, Madison, WI, USA) was used to produce a truncated, HIV-1 MA and CA (MACA) HaloTag^®^ fusion protein. MACA was amplified from pNL4-3, obtained from the NIH AIDS Research and Reference Reagent Program (Division of AIDS, NIAID, NIH) and then cloned into the pFC14K HaloTag^®^ vector, linearised with SgfI and EcoRI.

The pNL4-3MA-YFP-**∆***env* vector was used to generate pNL4-3MA-mCherry-Δ*env* [41]. Briefly, pNL4-3MA-YFP-Δ*env* was pretreated with ClaI to remove the yellow fluorescent protein gene, which replaced with the ClaI-treated mCherry gene.

### 2.2. Production of VSV-G-Pseudotyped Lentivirus

VSV-G-pseudotyped lentiviral vector particles in this study were produced in HEK293T cells, using the calcium phosphate co-transfection method. To produce VSV-G-pseudotyped ankyrin-EGFP viral particles, HEK293T cells were seeded on 10-cm dishes (3.5 × 10^6^ cells per dish), and co-transfected with the CGW transfer vector (10 µg/dish), the packaging construct pMDLg/pRRE (6.5 µg/dish), pRSV-Rev (2.5 µg/dish), and pMD.2G (3.5 µg/dish). Culture supernatant containing lentiviral vector particles were harvested at 24 and 48 h post-transfection, and concentrated by ultracentrifugation. The viral titers were determined by re-transduction of HEK293T cells with serial dilution of lentiviral vectors, and expressed as the percentage of EGFP positive cells determined by flow cytometry.

To produce VSV-G-pseudotyped HIV-1 Gag-mCherry virus particle. HEK293T cells were seeded on 10-cm dishes (6 × 10^6^ cells per dish), and co-transfected with pNL4-3MA-mCherry-∆*env* (7 µg/dish), pNL4-3∆*env* (3.3 µg/dish), and pMD.2G (4.5 µg/dish) using the TransIT-X2 transfection reagent (Mirus Bio, Madison, WI, USA), according to the manufacturer’s protocol. The VSV-G-pseudotyped lentiviral particles were harvested from the culture supernatant collected at 48 h. Virions were filtered through sterile syringe filters with a 0.45 µm Millex-HA filter (Merck Millipore, Burlington, MA, US). The high-titer virions stocks were prepared by ultracentrifugation at 100,000× *g*, at 4 °C, for 1.30 h. The viral pellets were resuspended in DMEM. The lentiviral titers were determined by transduction into HEK293T cells with serial dilution of the samples and expressed as the percentage of mCherry-positive cells determined by flow cytometer.

### 2.3. Establishment of HeLa Cells Stably Expressing Ankyrin

To generate HeLa cells stably expressing ankyrin, HeLa cells were transduced with the VSV-G-pseudotyped lentiviral vectors at MOI of 1 in a growth medium containing 8 μg/mL of Polybrene (Sigma Aldrich, Saint Louis, MO, USA). Each VSV-G-pseudotyped lentiviral vectors included VSVG–CGW–Myr (+) Ank^GAG^1D4-EGFP, VSVG–CGW–Myr (+) Ank^A3^2D3-EGFP. The transduced cells were then transferred to a humidified incubator, and maintained at 37 °C and 5% CO_2_ for 24 h. The cells were washed three times with a fresh growth medium and further cultured in fresh growth medium. The efficiency and stability of transduction were determined by fluorescence microscope and flow cytometer.

### 2.4. Analysis of Gag Distribution on the PM

To determine the distribution of Gag on the PM, HeLa cell monolayers (approximately 1 × 10^5^ cells/well in 24-well plates) were transfected with 1μg of plasmid, including pCEP4 plasmid carrying ankyrin-encoding gene (i.e., pCEP4-Myr (+) Ank^GAG^1D4-GFP, pCEP4-Myr (+) Ank^A3^2D3-GFP) and/or pFC14K-MACA HaloTag^®^, using the transIT-X2 transfection reagent (Mirus Bio, Madison, WI, USA). The cells were grown in humidified 5% CO_2_ atmosphere incubator at 37 °C for 48 h. MACA HaloTag^®^-expressing cells were stained with the HaloTag^®^ TMR Direct™ ligand (Promega, Madison, WI, USA) at a 1:1000 dilution for 16 h humidified 5% CO_2_ atmosphere incubator at 37 °C. The cells were then fixed with 4% paraformaldehyde and mounted using ProLong™ Diamond Antifade Mountant with DAPI (Thermo Scientific, Waltham, MA, USA). The fixed cells were viewed using a Leica TCS SP8 stimulated emission-depletion (STED) microscope. Raw images were iteratively deconvolved with Huygens software (Scientific Volume Imaging, Hilversum, The Netherlands).

### 2.5. Investigation of RNA Packaging and Gag Production

Transfection: 5 × 10^6^ cells (HEK293T cells) were seeded into wells and allowed to attach overnight. Two micrograms of pNL4-3∆*env* along with carrier DNA (pSP72 plasmid) or 1.6 µg (pNL4-3∆*env*) with or without pCEP4-(Myr+) Ankyrin-GFP (0.5:1 or 1:1 molar ratio with pNL4-3∆*env*) were co-transfected into HEK293T cell monolayers using the Lipofectamine™ 3000 Transfection Reagent (Thermo Scientific, Waltham, MA, USA), according to the manufacturer’s recommended procedure. Virion-containing supernatants were collected at 24 h post-transfection, centrifuged at for 10 min at 350× *g* and 4 °C, filtered (0.45 µm filter), and stored at −80 °C. The transfected cells were scraped off, extensively washed with cold PBS, centrifuged for 5 min at 350× *g* and 4 °C, and stored at −80 °C as a dried pellet.

Virion purification: Virions were purified from collected culture supernatants by ultracentrifugation through a 20% sucrose cushion at 100,000× *g* for 1.30 h at 4 °C. The pellets were resuspended in 160 µL of DMEM. Next, 25 µL of each virion sample was used for protein analysis, 125 µL was used for RNA extraction, and 10 µL was used for p24 quantification using a modified Genscreen^TM^ ULTRA HIV Ag-Ab Assay (Bio-Rad, Hercules, CA, USA).

RNA extraction: RNA from concentrated virus samples was extracted using the QIAamp Viral RNA Mini Kit in the presence of 5.6 µg of carrier RNA (Poly A) according to manufacturer’s recommended procedure (Qiagen, Hilden, Germany). The viral RNAs were then treated with DNase I (Thermo Scientific, Waltham, MA, USA) to eliminate the remaining DNA. Then, dried pellets containing 3 × 10^6^ cells were used for total cellular RNA extraction with a High Pure RNA Isolation Kit (Roche, Basel, Switzerland) in the presence of DNase, according to manufacturer’s recommended procedure.

Protein analysis: The remaining dried cell pellets were lysed to extract cellular protein using RIPA Lysis and Extraction Buffer (Thermo Scientific, Waltham, MA, USA), according to the manufacturer’s instructions. Total protein was measured using the Pierce BCA Protein Assay Kit (Thermo Scientific, Waltham, MA, USA). Proteins in 100 µg of cell lysate or 25 µL of concentrated virion were separated using SDS-PAGE and transferred to nitrocellulose membrane. HIV-1 Gag was detected with a mouse anti-CA antibody (hybridoma H183, NIH AIDS Reagent Program). Ankyrin protein expression was determined with a GFP Monoclonal Antibody. Horseradish peroxidase-conjugated (HRP) anti-mouse IgG (Seracare, Milford, MA, USA) was used as a secondary antibody. Cellular actin was detected using an HRP-conjugated mouse monoclonal anti-β-actin antibody (clone AC-15, Sigma-Aldrich, Saint Louis, MO, USA). ECL fluorescence was recorded using a ChemiDoc™ Touch Imaging System (Bio-Rad, Hercules, CA, USA).

Quantification of p24 in concentrated virions by performing p24 ELISA: The Genscreen^TM^ ULTRA HIV Ag-Ab Assay Kit (Bio-Rad, Hercules, CA, USA) was modified by replacing conjugate 1 with a biotinylated anti-capsid (CA) antibody and pre-incubating the sample with 10% Triton X-100 (virion lysis buffer, (Thermo Scientific, Waltham, MA, USA) at 37 °C for 30 min before beginning the manufacturer’s protocol.

RNA quantification: Reverse transcription (RT) was performed using the Transcriptor High Fidelity cDNA Synthesis Kit (Roche, Basel, Switzerland) with 1 µg of total cellular RNA sample or 0.5 µg of virion RNA. An oligo (dT) primer was used as an RT primer for viral RNAs and GAPDH mRNA, whereas specific internal primers were required for RT of 7SL and U6 RNA. After the RT reactions, the RT products were diluted, and quantitative polymerase chain reaction (PCR) experiments were run on a CFX96 Real-Time System (Bio-Rad, Basel, Switzerland). The RT products were amplified with 40 cycles of PCR: 95 °C for 15 s 58 °C for 12 s, and 72 °C for 20 s. The pair of primers (Table 1) were used at the final concentration of 0.5 mM in the real-time PCR for FL HIV-1 RNA, MS HIV-1 RNA, HIV-1 env RNA, GAPDH, 7SL, and U6. Quantification was performed using a standard curve comprised of 10^2^ to 10^9^ copies of the pNL4-3 plasmid.

### 2.6. Determination of an Effect of Ank^GAG^1D4 on HIV-1 Protease Activity

An effect of ankyrin on HIV-1 protease activity was determined using ELISA. The microtiter plate was coated with 5 µg/mL of recombinant H_6_MA-CA (His6-Matrix-Capsid) and incubated at 4 °C for 16 h. Then, the coated plate was washed with washing buffer 1 (0.05% Tween-20 in PBS) and blocked with 2% skim milk-PBS for 1 h. Next, plate was washed with washing buffer 1. The 5 µg/mL of recombinant Ank^GAG^1D4 or Ank^A3^2D3 (irrelevant ankyrin repeat protein) diluted in binding buffer (2% of BSA in 0.05% Tween 20-PBS) were added to coated well, then the reaction was placed at room temperature for 1 h. The H_6_MA-CA-binding activity of ankyrin was detected by rabbit anti-ankyrin polyclonal antibody (pre-absorbed with 5% of BSA in 0.05% Tween 20-PBS for 1 h) followed by goat anti-rabbit immunoglobulins-HRP (diluted in binding buffer). Besides, after 1 h incubation of Ankyrin to H_6_MA-CA coated plate, the HIV-1 protease activity was performed, the non-specific binding was washed with washing buffer 2 (1% Triton X100 (*v*/*v*) and 550 mM NaCl prepared in PBS, pH 7.4). The 6 mg/mL of soluble HIV-PR (diluted in binding buffer), while bacterial lysate BL21(D3) was used as a control, was added to the wells, and the plate was incubated at 37 °C for 1 h. Then, the wells were washed with washing buffer 2. The presence of the free C-terminus of MA after cleaving by HIV-PR was detected by an anti-MA (HB-8975) monoclonal antibody (specifically binds to the free C terminus of cleaved HIV-MA) followed by a goat anti-mouse immunoglobulins-HRP (diluted in binding buffer). The wells were then washed; followed by TMB substrate being added. The wells were then washed again with washing buffer 2 prior to adding SureBlue™ TMB Microwell Substrate (KPL) and the optical density at 450 nm (OD 450 nm) were measured after adding 1 N HCl.

### 2.7. Investigation of the Influence of Ank^GAG^1D4 on Organisation of CD81 during HIV-1 Assembly

HeLa cells or HeLa cells stably expressing ankyrin were transduced with VSV-G-pseudotyped HIV-1 Gag-mCherry at MOI of 2 in a growth medium containing 8 μg/mL of Polybrene (Sigma-Aldrich, MO, USA). Transduced HeLa cells were incubated in humidified 5% CO_2_ incubator. The excess lentiviruses were washed after 1 day of transduction. On the next following day, cells were collected to be analysed for CD81 expression using direct immunofluorescence staining and flow cytometry.

The direct immunofluorescence staining was performed in the adhered transduced HeLa cells on coverslip. Cells were washed with PBS and fixed with 4% paraformaldehyde-PBS for 10mins at room temperature. Cells were then washed twice with PBS and blocked with 10% human AB serum-PBS. Next, cells were stained with 100 µg/mL of Pacific Blue™ anti-human CD81 (Biolegend, San Diego, CA, USA). Finally, cells were washed and mounted with ProLong™ Gold Antifade Mountant (Thermo Scientific, Waltham, MA, USA). The CD81, Gag-mCherry, and Ankyrin-EGFP were visualised under a Nikon C2 plus confocal microscope. Excitation wavelengths were 405 nm for Pacific Blue, 488 nm for EGFP, and 560 nm for mCherry.

The transduced HeLa cells were also performed flow cytometry analysis to determine the surface CD81 expression by staining with 100 µg/mL of Pacific Blue™ anti-human CD81 (Biolegend, San Diego, CA, USA).

### 2.8. Statistical Analysis

Results are presented as the mean ± standard deviation (SD) of three independent assays. Statistical comparisons were determined by one-way analysis of variance (ANOVA) with GraphPad Prism software. Statistically significance was indicated as asterisk (*** *p* < 0.001; ** *p* < 0.01; * *p* < 0.1; ns, not significant).

## 3. Results

### 3.1. Ank^GAG^1D4 Affected Gag Production in HIV-1-Producer Cells

**Figure 1:**
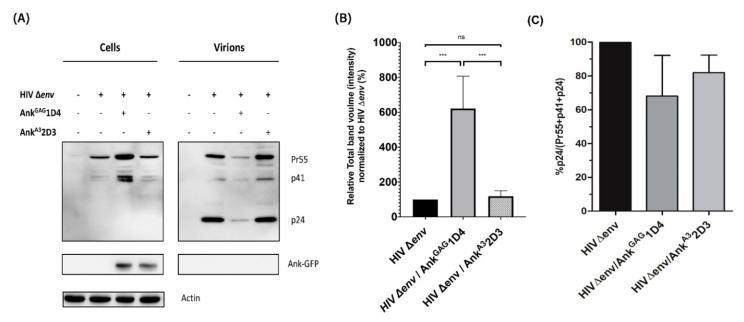
The effect of Ank^GAG^1D4 on Gag polyprotein levels inside Gag-producing cells and virions. (**A**) Representative western blot images of Gag in cells and virions released from HEK293T cell after 24 h co-transfected with an HIV-1 ∆*env* construct, with or without pCEP4-Myr (+) Ank^GAG^1D4-GFP or pCEP4-Myr (+) Ank^A3^2D3-GFP. Gag was detected using a mouse anti-CA antibody, and the expression of designed ankyrin repeat proteins was detected with an anti-GFP antibody. (**B**) A band-intensity histogram related to Gag proteins in cells was generated with Image lab software, and represented in a bar graph showing the relative intensity of Gag proteins normalised to HIV ∆*env* alone. (**C**) The percentage of HIV-1 Gag processing efficiency was calculated from the band intensity of HIV-1 Pr55, p41, and p24 in viral lysate using the p24/(pr55 + p41 + p24) formula. HEK293T cells transfected with pNL4-3 ∆*env* were used to normalise the data. Data represent the mean ± SD from the triplicate independent assay. *** *p* < 0.001; ns, not significant using one-way ANOVA.

HEK293T cells were co-transfected with pNL4-3∆*env* plasmid with or without pCep4-Myr (+) Ank^GAG^1D4-GFP or pCep4-Myr (+) Ank^A3^2D3-GFP (1:1 molar ratio with pNL4-3∆*env*). Proteins were extracted from cell pellets and concentrated virus samples, and analysed by western blotting with antibodies against CA, GFP, and β-actin. The results show that both Ank^GAG^1D4 and Ank^A3^2D3 (non-binder ankyrin control) were expressed in transfected cells as detected based on GFP expression but were not incorporated into virions (Figure 1A). The presence of both Ank^GAG^1D4 and Ank^A3^2D3 did not interfere with the Gag-processing pattern as Pr55Gag was processed to the p41 and p24 CA proteins. The accumulation of Pr55Gag and the p41 CA precursor was observed inside HEK293T cells transfected with the pNL4-3∆*env* and pCep4-Myr (+) Ank^GAG^1D4-GFP (Figure 1B). In contrast, in the presence of (Myr (+)) Ank^GAG^1D4, little or no virion release was observed, as shown by the strong decrease of p24 intensity in viral lysate (Figure 1A, B). Additionally, HIV-1 Gag processing efficiency from viral lysate was determined using image lab software (Figure 1C). The gag processing efficiency in the presence of (Myr+) Ank^GAG^1D4 was lower than the control cells. This evidence associates with the reduction in virion release. These results indicated that the intracellular expression of Ank^GAG^1D4 impaired virion release; thus, Gag accumulated inside the cells without affecting the Gag processing.

### 3.2. Ank^GAG^1D4 Is Not Associated with HIV-1 Protease Cleavage Site

**Figure 2:**
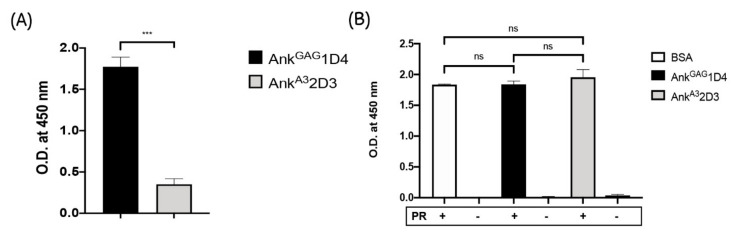
The effect of Ank^GAG^1D4 on HIV-1 protease activity. ELISA was performed to determine the effect of ankyrin protein on HIV-1 Gag processing by Protease. (**A**) The H_6_MA-CA-binding activity of artificial ankyrins. Plate was coated with recombinant H_6_MA-CA. Next, Ank^GAG^1D4 (Black bar) or Ank^A3^2D3 (Gray bar) was added to H_6_MA-CA coated well and incubate for 1 h. The H_6_MA-CA-binding activity was detected by adding rabbit anti-ankyrin polyclonal antibody followed by goat anti-rabbit immunoglobulins-HRP. The data presented in the bar graph was formerly normalised with no ankyrin control. (**B**) The activity of HIV-1 protease was determined using ELISA. Plate was coated with the target of HIV-1 protease (recombinant H_6_MA-CA). Prior to adding HIV-1 protease, the coated plate was incubated with BSA (White bar), Ank^GAG^1D4 (Black bar), or Ank^A3^2D3 (non-binding ankyrin protein) (Gray bar) for 1 h. The protease activity was determined by the presence of free C terminus of cleaved HIV-MA after cleaving with HIV-1 protease. The detection was measured by adding an anti-MA (HB-8975) monoclonal antibody (binds to the free C terminus of cleaved HIV-MA) followed by a goat anti-mouse immunoglobulins-HRP. The results represent as mean ± SD from a triplicate independent assay. *** *p* < 0.001; ns, not significant using one-way ANOVA.

According to Figure 1A, a few amounts of virion release and accumulation of Pr55Gag and the p41CA precursor inside the Gag-producing cells were observed, in the presence of Ank^GAG^1D4 as shown in Figure 1A. To confirm whether the binding of Ank^GAG^1D4 with HIV-1 capsid does not reside in the protease cleavage sites, Gag polyprotein processing by HIV-1 protease was determined using enzyme-linked immunosorbent assay (ELISA). In this experiment, the H_6_MA-CA recombinant protein, which contains the HIV-1 protease cleavage site between MA and CA, was used as a model. First, the specific binding of Ank^GAG^1D4 with coated H_6_MA-CA was determined. The result demonstrated the specific interaction of Ank^GAG^1D4 with coated H_6_MA-CA compared to Ank^A3^2D3 (Figure 2A).

Next, in order to exhibit that Ank^GAG^1D4 did not interfere with the HIV-1 protease cleavage site, Ank^GAG^1D4 was prior incubated with coated H_6_MA-CA for 1 h to ensure that the binding of Ank^GAG^1D4 with its target was achieved. Then, the HIV-1 protease was added to the bound H_6_MA-CA. The protease activity was measured by the presence of a free C-terminus of HIV-MA after cleaving with HIV-1 protease. The result confirmed that the binding of Ank^GAG^1D4 with H_6_MA-CA did not interfere with the HIV-1 protease cleavage site as detected by free C-terminus of HIV-MA after cleaving with HIV-1 protease at the same levels as Ank^A3^2D3 and control (Figure 2B).

### 3.3. Ank^GAG^1D4 Impaired Gag Distribution at the PM

**Figure 3:**
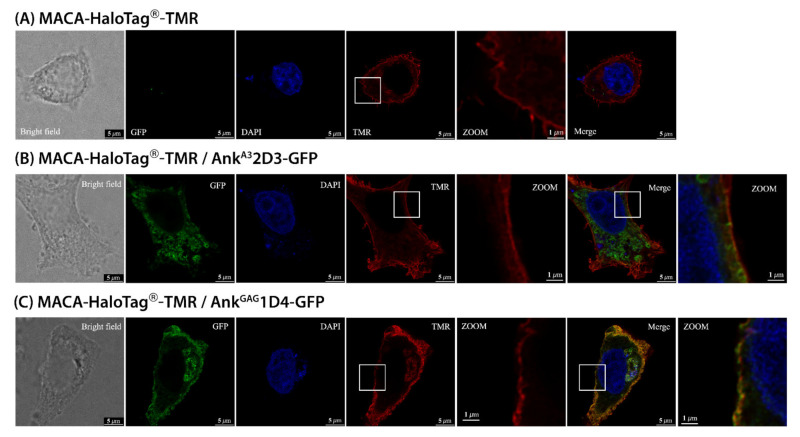
Co-expression of Ankyrin-GFP proteins and the MACA-HaloTag^®^ protein in Hela cells. Gag distribution at the membrane of Hela or HeLa cells expressing ankyrin was visualised by STED microscope. (**A**) Hela cells were transfected with pFC14K-MACA-HaloTag^®^ alone, (**B**) co-transfected with pCep4-Myr (+) Ank^GAG^1D4-GFP and pFC14K-MACA HaloTag^®^, or (**C**) co-transfected with pCep4-Myr (+) Ank^A3^2D3-GFP and pFC14K-MACA HaloTag^®^. After 48 h incubation, cells were stained with the HaloTag^®^ TMR Direct™ ligand, then mounted with ProLong™ Diamond Antifade Mountant with DAPI. The white squares indicate areas of interest in the PM of HeLa cells that are shown at higher zoom levels. The data shown in this figure is a representative of several imaged fields. Green represents GFP-tagged ankyrin expression in HeLa cells. Blue is the nucleus, and red indicates MACA- HaloTag^®^ protein.

As viral release was impaired by Ank^GAG^1D4, we were interested in determining which steps of virus assembly were affected. In this experiment, HIV-1 Gag distribution in ankyrin-expressing HeLa cells was demonstrated by stimulated emission depletion (STED) microscopy. HeLa cells were transfected with the pFC14K-MACA-HaloTag^®^ plasmid with or without pCEP4-Myr (+) Ankyrin-GFP. After transfection, MACA-HaloTag^®^ expression was traced by staining with the HaloTag^®^ TMR Direct™ ligand. As expected, the MACA-HaloTag^®^ localised mainly at the plasma membrane (Figure 3A). When co-expressing MACA-HaloTag^®^ and Myr + Ank^A3^2D3 (non-binder ankyrin control) little colocalisation was observed (Figure 3B), whereas MACA-HaloTag^®^ colocalised with Myr (+) Ank^GAG^1D4 throughout the PM (Figure 3C). Interestingly, in the zoomed-in view images (Figure 3C), the MACA HaloTag^®^-expression pattern on the PM of Myr (+) Ank^GAG^1D4 expressing HeLa cells was characterised by dispersion all over the cell periphery as small, punctate dots. This pattern was totally different from that observed in HeLa cells expressing MACA-HaloTag^®^ alone or MACA-HaloTag^®^ and Ank^A3^2D3, which showed smooth distribution throughout the PM (Figure 3A,B, respectively). These observations showed that Ank^GAG^1D4 disturbed the Gag distribution, suggesting the possibility that Ank^GAG^1D4 disrupted the Gag polymerisation process.

### 3.4. Ank^GAG^1D4 Affected RNA Packaging

**Figure 4:**
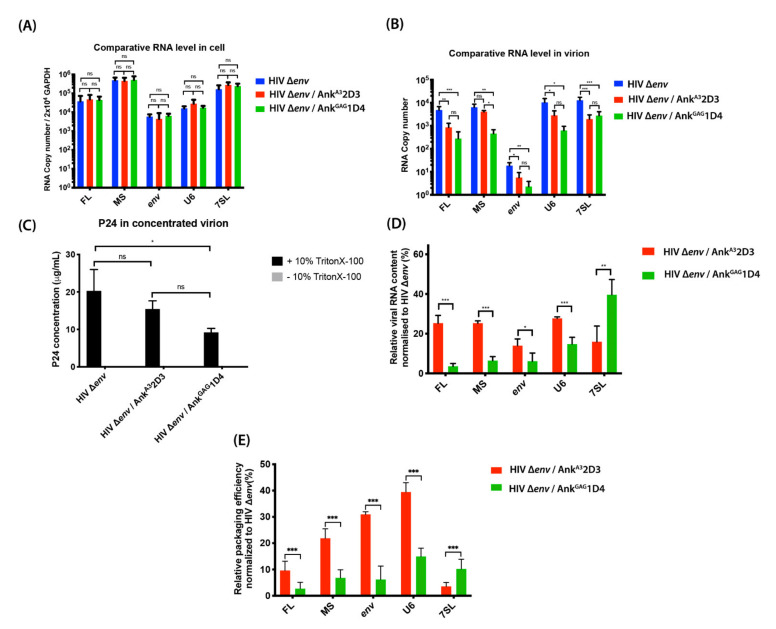
RNAs levels and packaging efficiency. At 24 h post-transfection, (**A**) Total cellular RNA was extracted and purified. The amounts of viral RNA (FL, MS, and env) and cellular RNA (U6 and 7SL) within cells were determined by RT-qPCR. The copy numbers were normalised to that of cellular GAPDH mRNA. (**B**) Total RNA in virions was concentrated, and the intravirion levels of viral RNA (FL, MS, and env) and cellular RNA (U6 and 7SL) were determined by qPCR. (**C**) The p24 concentrations in virion-containing supernatants were determined in the presence or absence of 10% TritonX-100, using a p24-ELISA. (**D**) The viral RNA contents were calculated by the percentage of total RNA level in virions at 100 ng of p24 (RNA copies/100 ng p24 × 100) normalised to HIV ∆*env* alone. (**E**) The packaging efficiency was calculated by the total viral RNA in virions relative to total cellular RNA and normalised to HIV ∆*env* alone. Data represent the mean ± SD from the triplicate independent assay. *** *p* < 0.001; ** *p* < 0.01; * *p* < 0.1; ns, not significant using one-way ANOVA.

HIV-1 Gag specifically recognises the FL RNA dimer in the cytosol and traffics to the PM as a Gag–FL RNA complex to join Gag-containing particles and displace RNA-containing particles at assembly sites [4]. Additionally, other viral RNAs (MS, env) and cellular RNAs can be packaged into the virion. Since Ank^GAG^1D4 modifies the Gag pattern at the PM, we tested whether Ank^GAG^1D4 could also affect FL RNA packaging into new viral particles. To do so, HEK293T cells were co-transfected with pNL4-3∆*env* and pCEP4-Myr (+) Ankyrin (Ank^GAG^1D4 or Ank^A3^2D3)-GFP (0.5:1 molar ratio with pNL4-3∆*env*). After 24 h post-transfection, cells and virions were collected, then subjected to RNA extraction. RNA samples were studied by RT-qPCR analysis of viral (FL, MS, and env) and cellular (GAPDH, 7SL, and U6) RNAs. The result indicates that Ank^GAG^1D4 and Ank^A3^2D3 did not affect the levels of viral and cellular RNAs in transfected cells (Figure 4A). The molar ratio of pNL4-3 ∆*env* to pCEP4-Myr (+) Ankyrin at 1:1 was not appropriate since the amount of generated virion was not sufficient for further analysis (Figure 1A). we diminished the inhibitory effect of Ank^GAG^1D4 in HIV-1 transfected cells, the molar ratio of pNL4-3∆*env* to pCEP4-Myr (+) Ankyrin was adjusted to 2:1 for sufficient virions collected to analyse their RNA contents by RT-qPCR. The results showed that both Ank^GAG^1D4 and Ank^A3^2D3 decreased the intravirion levels of viral RNAs (FL, MS, and env) and cellular RNAs (U6 small nuclear RNA (snRNA) and 7SL), compared to HEK293T cells expressing HIV-1 ∆*env* alone (Figure 4B). Ank^GAG^1D4 reduced the intravirion viral and cellular RNA levels much more than Ank^A3^2D3 (except for 7SL RNA; Figure 4B).

Additionally, viral release was measured by p24 ELISA. A control experiment was systematically performed without 10% Triton X-100 to control for the absence of cellular p24 contamination. As shown in Figure 4C, Ank^GAG^1D4 decreased the production of viral progeny, even though it was not totally abolished because we adjusted the inhibitory effect of Ank^GAG^1D4, as described above. Notably, without 10% Triton X-100, the p24 levels in all samples were undetectable, indicating that the p24 levels measured corresponded to intravirion p24. An equal number of viral particles (100 ng of p24) was subjected to compensate for the level of VLP-associated Gag. The level of differential RNA contents from the same number of virions was analysed. Both ankyrins negatively reduced the viral RNA contents (Figure 4D) and affected the packaging of both viral RNAs and cellular RNAs (Figure 4E). Interestingly, in comparison to Ank^A3^2D3, Ank^GAG^1D4 drastically decreased RNA packaging (Figure 4E). Especially for the incorporation of FL HIV-1 RNA, which reflected the indispensable for infectious virion. However, Ank^GAG^1D4 promoted significantly higher incorporation of 7SL cellular RNA into viral particles than did Ank^A3^2D3. This result indicated that viral particles released from the Ank^GAG^1D4-Gag producing cells lost their potential to encapsidate viral RNAs and cellular RNAs.

### 3.5. Ank^GAG^1D4 Restored CD81 Tetraspanin Localisation at the PM in Gag-Expressing Cells

**Figure 5:**
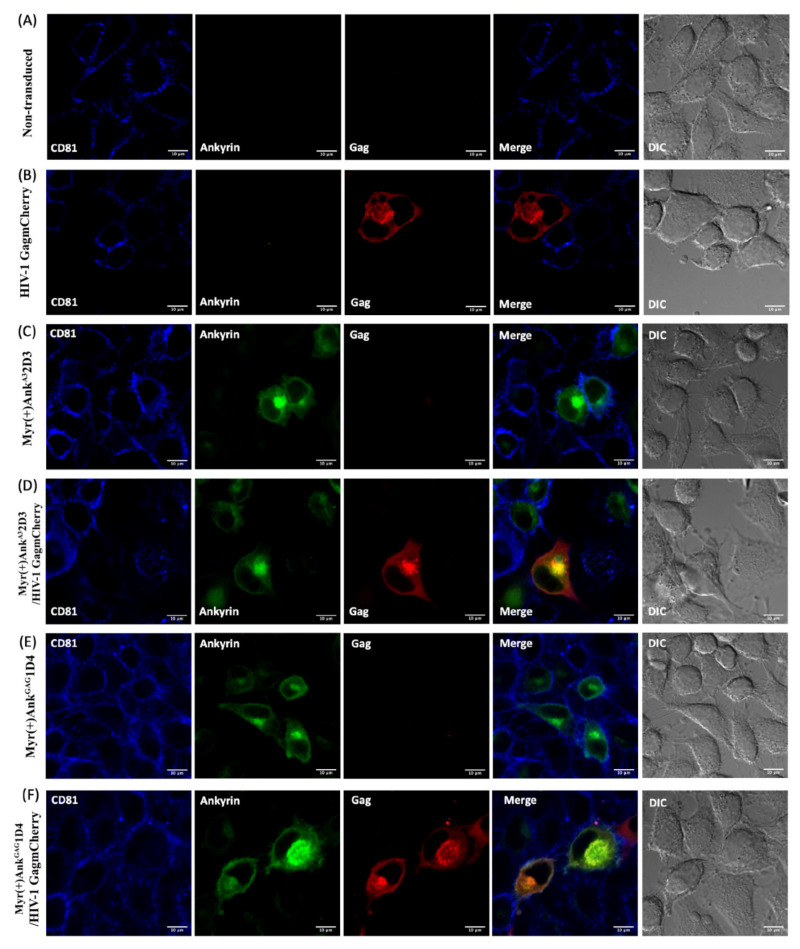
CD81 tetraspanin organisation at the PM during HIV-1 release. HeLa or HeLa cells stably expressing ankyrin (EGFP) were transduced with VSV-G-pseudotyped HIV-1 GagmCherry. After 48 h, cells were harvested, and stained with Pacific Blue™ anti-human CD81. CD81 tetraspanin organisation was visualised under conventional confocal microscopy. (**A**) Non-transduced HeLa cells, (**B**) Transduced HeLa cells, (**C**) HeLa cells stably expressing Myr (+) Ank^A3^2D3, (**D**) Transduced HeLa cells stably expressing Myr (+) Ank^A3^2D3, (**E**) HeLa cells stably expressing Myr (+) Ank^GAG^1D4, (**F**) and transduced HeLa cells stably expressing Myr (+) Ank^GAG^1D4. Scale bar: 10 µm. The image shown in this figure is a representative data of several imaged fields. Blue, green and red represent CD81 tetraspanin, EGFP-tagged ankyrin, and GagmCherry, respectively.

During HIV-1 assembly and release, the tetraspanins transmembrane glycoprotein form dynamic networks with other proteins and HIV-1 proteins including HIV-1 Gag and Env at the PM [13,18,19]. Additionally, CD81 and CD9 tetraspanins expression on the PM were found to decrease during viral release [14] and were incorporated with released virions [18,22]. For this reason, we hypothesised that the distortion of Gag polymerisation by Ank^GAG^1D4 could affect the CD81 tetraspanin remodelling at the PM. To address this issue, CD81 tetraspanins remodelling was assessed using conventional confocal microscopy. In this experiment, HeLa cells and ankyrin-expressing HeLa cells were transduced with VSV-G pseudotyped HIV-1 GagmCherry. At 48 h post-transduction, immunostaining was performed to demonstrate the distribution of CD81 tetraspanins under confocal microscopy. In this experiment, as Ank^GAG^1D4 influenced the HIV-1 Gag distribution at the PM (Figure 3C), tetraspanin recruitment to virus-assembly sites was considered. CD81 tetraspanin distribution in the PM of HeLa and ankyrin expressing HeLa cells was initially determined using confocal microscopy. Regarding confocal imaging, surface expression of CD81 remained with Myr (+) Ank^GAG^1D4-EGFP or Myr (+) Ank^A3^2D3-EGFP expressing HeLa cells (Figure 5C,E, respectively).

**Figure 6:**
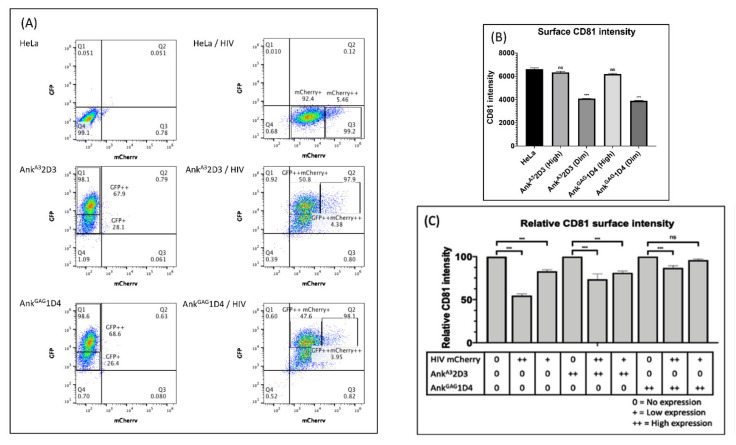
CD81 tetraspanin surface intensity and viral production. HeLa cells and HeLa cells stably expressing Myr (+) Ank^GAG^1D4-GFP or Myr (+) Ank^A3^2D3-GFP were transduced with VSV-G-pseudotyped HIV-1 GagmCherry. (**A**) After 48 h, the level of ankyrin expression and HIV-1 GagmCherry expression was analysed by flow cytometry. Populations were defined based on their fluorescence intensity ((+) = intermediate expression, (++) = high expression). (**B**) Surface CD81 expression on non-transduced cells was measured by flow cytometry. The intensity of CD81 on non-transduced HeLa and ankyrin-expressing HeLa cells was shown in Bar graph. (**C**) Surface CD81 expression on transduced HeLa and ankyrin-expressing HeLa cells was determined by flow cytometry. Relative surface CD81 intensity was calculated by the percentage of surface CD81 intensity of transduced cells and normalised against surface CD81 intensity of non-transduced cells. Data represent the mean ± SD from triplicate independent assay. Significant differences (*** *p* < 0.001; ns, not significant) were determined between transduced and non-transduced HeLa cells or ankyrin-expressing HeLa cells using one-way ANOVA. Ank^GAG^1D4, Ank^A3^2D3 represent HeLa cells stably expressing Myr (+) Ank^GAG^1D4-EGFP and Myr (+) Ank^A3^2D3-EGFP, respectively.

Additionally, the level of overall surface CD81 expression in ankyrin-expressing cells was confirmed by flow cytometry. Accordingly, high expression levels of Ankyrin protein did not disturb the expression of CD81 at the PM (Figure 6B). Therefore, the high-expressing Ankyrin protein was used in further study. Next, we analysed the surface expression of CD81 on HeLa cells expressing Gag-mCherry. Most of the HeLa transduced cells were positively expressed Gag-mCherry and were defined in 2 populations: cells with intermediate (mCherry+) and high (mCherry++) levels of Gag-mCherry expression (Figure 6A). After staining with anti-human CD81, the surface CD81 expression disappeared on PM of HeLa cells expressing Gag-mCherry (Figure 5B) and the CD81 intensity was decreased by 50% in cells strongly expressing Gag-mCherry as compared to the non-transduced cells (Figure 6C). This result agreed with another study showing that CD9 and CD81 tetraspanin levels decreased in the PM at 48 h post-transfection in HeLa cells expressing Gag–GFP (14). Surprisingly, introducing HIV-1 Gag–mCherry into HeLa cells strongly expressing Ank^GAG^1D4 cells efficiently maintained the CD81 tetraspanin on the PM up to 95% in the intermediate expression of Gag-mCherry and 86% in the high expression of Gag-mCherry (Figure 6C). Nevertheless, the 80% (in the intermediate expression of Gag-mCherry) and 70% (in the high expression of Gag-mCherry) surface CD81 restoration were observed in HIV-1 Gag-mCherry transduced HeLa cells stably expressing Ank^A3^2D3 (Figure 6C). This phenomenon reflects the effect of the N-terminus myristoyl group of AnkA32D3 inserted into the inner leaflet of the cell membrane leading to nonspecifically disturbing of HIV-1 Gag assembly. Comparing the efficient restoration of surface CD81 between Ank^GAG^1D4 and Ank^A3^2D3 in HIV-1 Gag-mCherry transduced cells, the Ank^GAG^1D4 was significantly sustained the CD81 distribution at the PM in both cells with intermediate (*p* < 0.001) and high (*p* < 0.001) levels of Gag-mCherry expression than Ank^A3^2D3. These findings indicated that disturbing Gag distribution at PM by Ank^GAG^1D4 impaired membrane remodelling during virus egress.

## 4. Discussion

Ank^GAG^1D4 is a designed ankyrin repeat that binds a conserved sequence in the N-terminal region of the HIV-1 CA protein. Ank^GAG^1D4 exhibits intracellular antiviral activity in the viral assembly process [37,38]. Recently, Ank^GAG^1D4 exerts broad-spectrum antiviral activity by blocking the assembly of chimeric NL4-3-based virions derived from circulating strains among northern Thai patients [39]. Additionally, an affinity-improved Ank^GAG^1D4 successfully demonstrated the enhancement of anti-HIV-1 activity in HIV-1 NL4-3 and HIV-1 maturation inhibitor-resistant (MIR) virus [42]. Although Ank^GAG^1D4 performs a potent anti-HIV activity in HIV-1 infected cells, more understanding of molecular mechanisms which impaired HIV-1 production is required.

In this study, we showed that Ank^GAG^1D4 did not interfere with the protease cleavage site, since the accumulation of the Pr55 Gag precursor and p41 proteins was observed inside the cells. Moreover, the CA p24 protein (the final product of Gag maturation) was detected in relatively few viral particles released from cells expressing Ank^GAG^1D4. Moreover, this phenomenon was confirmed by directly detecting the free C-terminus epitope of cleaved HIV-MA generated by HIV-1 protease-cleaved H_6_MACA. This epitope was specifically captured by monoclonal antibody HB-8975 [43]. This data indicated that the interaction of Ank^GAG^1D4 to HIV-1 CA did not hinder or alter the HIV-1 protease cleavage site on recombinant H_6_MACA.

The late stages of the HIV-1 life cycle include many events (such as intracellular trafficking, assembly, budding, release, and maturation) in which Gag plays a key role. Disturbing these processes diminishes the production of HIV-1 infectious particles. Inside Gag-producing cells, PI (4,5) P2 phospholipids interact with the HBR of MA and allosterically promote the release of sequestered myristate into lipid bilayers (2,3). The cytosolic, compact shape of Gag can be converted to an extended form and form polymers with other Gag proteins in the extended form [44]. Previously, using conventional pinhole confocal microscopy, the specific colocalisation of Myr (+) Ank^GAG^1D4 and HIV-1 Gag at the PM of infected cells was observed [38]. Unexpectedly, Gag colocalisation with Myr (+) Ank^A3^2D3, which does not bind to HIV-1 Gag, was also observed. Due to the diffraction limit of the light used for conventional confocal microscopy, it is not possible to distinguish spots less than 200 nm apart [45]. Thus, in this study, STED was used to overcome this limitation and monitor Gag distribution pattern at the PM. Previously, STED was employed to visualise the distribution of the Env glycoprotein on the surface of HIV-1 particles [46]. In addition, STED was recently used to demonstrate that HIV-1 Gag specifically restricts PI (4,5) P2 and cholesterol at assembly sites [47]. Using STED microscopy, we clearly observed that the MACA-HaloTag^®^ distributed continuously at the PM of MACA-HaloTag^®^ expressing cells. However, the continual pattern of MACA-HaloTag^®^ was clearly impaired in the presence of Ank^GAG^1D4 (Figure 3C). The apparent visualisation of dark gaps on the cell surface implied that embedding of Ank^GAG^1D4 in the PM probably influenced the Gag polymerisation (Figure 3C). To prove this, the electron microscopic (EM) analysis should be applied to assure the disturbance of MACA-HaloTag^®^ at the plasma membrane resulting in the disruption of CA lattice formation in defective viral progeny. Noticeably, the efficiency of HIV assembly was disrupted resulting in the reduction of viral progeny. Even though Ank^GAG^1D4 efficiently reduces viral production, the effects of Ank^GAG^1D4 on viral budding and membrane scission through the ESCRT machinery should be further investigated. ESCRT is not only associated with viral budding, but also participates in MVB biogenesis, which is important for transporting ubiquitinated protein for degradation [48]. As shown in Figure 3B, Myr + Ank^A3^2D3 mainly localised in the cytoplasm. We inferred the involvement of the ESCRT degradation pathway, since Gag was not the specific target of Myr + Ank^A3^2D3. Furthermore, the indirect disturbance of Gag-Gag multimersation by maturation inhibitor, bevirimat, caused abnormal virion morphology [49]. Likewise, the aberrant virion morphology caused by other CA, assembly, or maturation inhibitors has been well characterized and described by using electron microscopy [50,51,52]. Altering this critical step of HIV assembly by Ank^GAG^1D4 possibly causes aberrant virion production. Investigation of the alteration of virion morphology produced by Ank^GAG^1D4 expressing cells using EM should be further evaluated.

Because Ank^GAG^1D4 did not significantly disturb the cellular FL HIV-1 RNA level, an effect on HIV-1 RNA transcription was excluded. Therefore, accumulation of the Gag polyprotein inside Ank^GAG^1D4 expressing cells resulted from the retardation of the Gag polyprotein-release rate during the assembly process. As the extended Gag conformation generally uses the NC domain to recruit FL RNA and initiate the viral-assembly stage [53]. RNA levels in virions and RNA-packaging efficiency have been studied by RT-qPCR (reviewed in [54]). In this study, Gag clearly lost its ability to specifically incorporate FL HIV-1 RNA into virions in the presence of Ank^GAG^1D4 (Figure 4D,E). The ability of Gag to multimerize on RNA via the NC domain is important for RNA packaging. Since NC is not the target of Ank^GAG^1D4, the mechanism of Ank^GAG^1D4 should rather participate in the disturbance of Gag multimerisation. The networking of Gag is crucial for successfully stabilizing RNA at the plasma membrane in the preassembly stage [53,55]. According to STED analysis, the impairment of Gag distribution in Ank^GAG^1D4 expressing cells was indicated, thus, reflecting the interference of the Gag polymerisation process. In general, FL HIV-1 RNA and spliced RNA can be packaged into virions due to the presence of an internal loop and the lower part of SL1 in the 5′-untranslated region of HIV-1 RNA. However, FL HIV-1 RNA is preferably selected by the Gag precursor with higher affinity than spliced RNA due to the counterbalance-regulation domain spanning nucleotides downstream of SL4 and upstream of SL1, and the specific region between nucleotides 355–400, which is not present in spliced RNA [8,10]. Accordingly, the presence of Ank^GAG^1D4 also interfered with the incorporation of spliced RNAs (MS and env RNA) and cellular RNAs (U6 snRNA and 7SL) into nascent virions. Data from several studies showed that HIV-1 particles can specifically package small Pol-III transcripts in addition to FL RNA [11]. Ank^GAG^1D4-Gag expressing cells not only lost HIV-1 RNA encapsidation, but also cellular RNA incorporation (Figure 4D,E). Compared to Ank^A3^2D3, the FL RNA reduction induced by Ank^GAG^1D4 was accompanied by increased 7SL RNA incorporation into virions (Figure 4D,E). Interestingly, these opposing effects on the FL and 7SL RNA packaging levels were also reported when the packaging signal was deleted from FL RNA [9]. Additionally, increasing 7SL RNA incorporation in virions was possibly caused by Gag accumulation induced by Ank^GAG^1D4, since Gag accumulation inside producer cells previously promoted 7SL RNA incorporation into virions [56]. A partial reduction of the RNA-packaging efficiency was also observed in the presence of Ank^A3^2D3, which may have been caused by competition between the myristoyl group of Ank^A3^2D3 and Gag in recruiting cholesterol molecules [57]. In contrast to Ank^A3^2D3, a tremendously impaired RNA packaging in Ank^GAG^1D4 expressing cells was significantly observed. Additionally, it was clear that the alignment of Gag at PM in Ank^A3^2D3-Gag-expressing cells was not disturbed (Figure 3B), as Gag is not the specific target of Ank^A3^2D3. Thus, RNA incorporation partially occurred in the presence of Ank^A3^2D3.

The clustering of tetraspanins with Gag and Env at the PM has been observed in diverse types of HIV-1 infected cells [18,58]. Gag polymerisation at the PM can induce the formation of microdomains, including TEMs [59]. TEMs have been proposed as gateways for HIV-1 assembly and budding [14,60]. We observed that at 48 h post-transduction with VSV-G-pseudotyped HIV-1 GagmCherry, the CD81 tetraspanin vanished from the PM. Diminishing PM expression of the CD81 tetraspanin in HIV-1-infected cells was related to VLP release [14]. Interestingly, we also observed that inhibiting HIV-1 egress with Ank^GAG^1D4 caused a rebound of CD81 tetraspanin on the PM of HIV-1 Gag-expressing cells. These data indicated that Ank^GAG^1D4 caused membrane remodelling during viral release, since Gag polymerisation was disturbed. Beyond Ank^GAG^1D4, no other small molecules or peptide inhibitors targeting CA have been reported to affect the remodelling of tetraspanins on the PM. Moreover, the presence of tetraspanins at exit sites can reduce syncytial formation in virus-producing cells and cell-to-cell fusion induced by the virus [61,62]. Because Ank^GAG^1D4 is capable of preventing syncytial formation [37,38,39], it is worth further investigating its utility in blocking viral transfer via cell-to-cell contact.

## 5. Conclusions

The data obtained in this study illustrate the molecular mechanisms whereby Ank^GAG^1D4 blocks HIV replication. The intracellular expression of Ank^GAG^1D4 disrupted the primary checkpoint at the late stages of viral progeny production when the Gag polyprotein formed. The resulting intracellular accumulation of the Gag polyprotein was followed by inefficient Gag assembly and decreased RNA packaging. The disruption of CD81 tetraspanin membrane remodelling is proposed as a significant marker of defective HIV replication. Taken together, these findings could influence future strategies for using the altered scaffold protein as an anti-HIV-1 molecule.

## Figures and Tables

**Table 1:** List of primer used in RT-qPCR.

Target RNA	Primer Name	Primer Sequence
FL HIV-1 RNA	sHIV-1306	5′-TCAGCATTATCAGAAGGAGCCACC-3′
	aHIV-1541	5′-TCATCCATCCTATTTGTTCCTGAAG-3′
MS HIV-1 RNA	sHIV-5967	5′-CTATGGCAGGAAGAAGCGGAG-3′
	aHIV-8527	5′-CAAGCGGTGGTAGCTGAAGAG-3′
HIV-1 env RNA	sHIV-729SD1A5	5′-GAGGGGCGGCGACTGGAAGAA-5′
	aHIV-6134	5′-ACTATGGACCACACAACTATTGC-5′
GAPDH	GA-721	5′-GCTCACTGGCATGGCCTTCCGTGT-5′
	GA-931	5′ TGGAGGAGTGGGTGTCGCTGTTGA-5′
7SL	s7S-22	5′-CTGTAGTCCCAGCTACTCG-5′
	a7S-148	5′-CCCGGGAGGTCACCATATT-5′
U6	sU6-3	5′-GCTCGCTTCGGCAGCACATATACT-5′
	aU6-103	5′-TATGGAACGCTTCACGAATTTGCG-5′

## Data Availability

Data are contained within the article.
